# Characteristics of urinary and serum soluble Klotho protein in patients with different degrees of chronic kidney disease

**DOI:** 10.1186/1471-2369-13-155

**Published:** 2012-11-23

**Authors:** Tetsu Akimoto, Hiromichi Yoshizawa, Yuko Watanabe, Akihiko Numata, Tomoyuki Yamazaki, Eri Takeshima, Kana Iwazu, Takanori Komada, Naoko Otani, Yoshiyuki Morishita, Chiharu Ito, Kazuhiro Shiizaki, Yasuhiro Ando, Shigeaki Muto, Makoto Kuro-o, Eiji Kusano

**Affiliations:** 1 Division of Nephrology, Department of Internal Medicine, Jichi Medical University, 3311-1 Yakushiji, Shimotsuke-Shi, TOCHIGI, 329-0498, Japan; 2 Department of Pathology, University of Texas Southwestern Medical Center, 6000 Harry Hines Blvd., Dallas, TX, 75390-9072, USA

**Keywords:** Klotho, Chronic kidney disease, Renal function, Urinary protein, Creatinine

## Abstract

**Background:**

Klotho is a single-pass transmembrane protein, which appears to be implicated in aging. The purpose of the present study was to characterize the relationship between the soluble Klotho level and renal function in patients with various degrees of chronic kidney disease (CKD).

**Methods:**

The levels of soluble Klotho in the serum and urine obtained from one hundred thirty-one CKD patients were determined by a sandwich enzyme-linked immunosorbent assay system.

**Results:**

The amount of urinary excreted Klotho during the 24 hr period ranged from 1.6 to 5178 ng/day (median 427 ng/day; interquartile range [IR] 56.8-1293.1), and the serum Klotho concentration ranged from 163.9 to 2123.7 pg/ml (median 759.7 pg/ml; IR 579.5-1069.1). The estimated glomerular filtration rate (eGFR) was significantly correlated with the log-transformed values of the amount of 24 hr urinary excreted Klotho (r = 0.407, p < 0.01) and the serum Klotho levels (r = 0.232, p < 0.01). However, a stepwise multiple regression analysis identified eGFR to be a variable independently associated only with the log-transformed value of the amount of 24-hr urinary excreted Klotho but not with the log-transformed serum Klotho concentration. Despite the strong correlation between random urine protein-to-creatinine ratio and the 24 hr urinary protein excretion (r = 0.834, p < 0.01), a moderate linear association was observed between the log-transformed value of the amount of 24 hr urinary excreted Klotho and that of the urinary Klotho-to-creatinine ratio (Klotho/Cr) in random urine specimens (r = 0.726, p < 0.01).

**Conclusions:**

The amount of urinary Klotho, rather than the serum Klotho levels, should be linked to the magnitude of the functioning nephrons in CKD patients. The use of random urine Klotho/Cr as a surrogate for the amount of 24-hr urinary excreted Klotho needs to be evaluated more carefully.

## Background

Klotho is a single-pass transmembrane protein with a long extracellular domain and a short cytoplasmic tail, which appears to be implicated in aging, and regulates both the mineral transport and signaling mediated by fibroblast growth factor 23 (FGF23) [1,2]. The kidneys, parathyroid gland, and choroid plexus of the brain have been identified on as the sites where the Klotho is predominantly expressed, while the extracellular domain of Klotho may be cleaved and released into the blood, cerebrospinal fluid, and urine, as a soluble form [2-5]. Recently, we have shown that the total amount of urinary excreted soluble Klotho is correlated with the residual renal function among patients with advanced chronic kidney disease (CKD) [6]. However, the qualitative and quantitative analyses regarding the soluble Klotho in the subjects with different stages of CKD have been insufficient. To remedy this, we performed a cross-sectional study to characterize the level soluble Klotho in patients with various degrees of chronic renal dysfunction. The impact of accompanying renal characteristics on the soluble Klotho level was also investigated.

## Methods

Between June 2011 and February 2012, 131 ambulatory CKD patients with different stages of disease were recruited and enrolled in the present study. The stages of CKD were determined by the estimated glomerular filtration rate (eGFR) based on the abbreviated Modification of Diet in Renal Disease (MDRD) Study equation with a Japanese coefficient, calculated by the eGFR (ml/min/1.73 m^2^) = 0.881 × 186 × Age ^–0.203^ × serum creatinine ^-1.154^ (if female × 0.742) [7,8]. Patients receiving renal replacement therapy were excluded from the study. All subjects were being followed at the renal unit of Jichi Medical School Hospital and were stable. None were critically ill at the time of the study. The patients’ usual medications, such as anti-hypertensive agents, erythropoietin, diuretics, oral active vitamin D sterols including calcitriol and alfacalcidol, and CaCO_3_ as a phosphate binder, were continued during the study period. The research protocol was approved by the Medical Ethics Committee of Jichi Medical University, and all subjects included in the present study provided their informed consent.

After a sample of each urine specimen voided during a 24 hr period was collected, random urine samples were obtained when a blood sample for the study was also collected. Both the urine and blood sample were collected in the morning. The total protein, albumin, sodium, chloride, potassium, calcium, inorganic phosphate, urea, and creatinine levels in the serum were all measured just after the collection. The volume, total protein, albumin, sodium, chloride, potassium, calcium, inorganic phosphate, urea, and creatinine were measured in the 24-hour urine collection, and the same parameters were also evaluated in the random urine collections. The soluble Klotho concentrations were determined in each sample by a solid-phase sandwich enzyme-linked immunosorbent assay (ELISA) kit (Immuno-Biological Laboratories, Gunma, Japan) according to the manufacturer’s protocol [6,9]. The minimum level of detectability of the assay was 6.15 pg/ml, and the minimum level was below the serum and urine concentrations that were found in the present study. The intra- and inter-assay coefficients of variation were <10% [9]. The serum FGF23 levels were determined using a sandwich ELISA kit (Kainos Laboratories, Tokyo, Japan). The inter-assay variation in the normal and elevated concentration range was less than 6% [10]. An electrochemiluminence immunoassay for determining the serum intact parathyroid hormone (iPTH) levels and a double antibody radioimmunoassay for identifying the serum calcitriol levels were performed by a commercially based clinical diagnostic testing service (SRL, Inc., Tokyo, Japan). The serum soluble Klotho concentration or the amount of urinary soluble Klotho was normalized by a common logarithm (Log) when a reduction of skewness was required.

The data are expressed as either the number of participants or as the percentage (%) of the study population. The remaining data are expressed as the means ± SD, or medians and interquartile range (IR) for variables of a skewed distribution. The groups were compared using a one-way analysis of variance with Dunnet’s method for normal distributions and the Kruskal–Wallis test with Dunn’s method for skewed distributions. Categorical variables were assessed using the chi-square test with nonparametric post-hoc pairwise comparisons [11,12]. The relationships between the CKD stages and the amount of 24 hr urinary excreted Klotho or the serum levels of Klotho were assessed by Spearman’s correlations (Spearman’s rho), while the various linear relationships were assessed by Pearson’s product moment correlation if deemed necessary. A stepwise forward multiple regression analysis was conducted considering soluble Klotho as a dependent variable, and the eGFR, age, amount of 24 hr urinary excreted protein, serum total protein, serum albumin, serum phosphorus, serum iPTH, and serum calcitriol levels were included in the statistical model. This method was also applied to evaluate the significance of the soluble urinary Klotho level, including the 24 hr urinary excreted Klotho and random urinary Klotho-to-creatinine ratio, on eGFR as an independent predictive variable. The F-values for the inclusion and exclusion of variables was set at 4.0. The differences between the independent correlation coefficients were compared statistically by performing a Fisher’s r-to-z transformation [13-15]. P-values <0.05 were considered to be statistically significant. The statistical analyses were performed using the SigmaPlot software program 11 for Windows (Systat Software, Inc., San Jose, CA) unless otherwise stated.

## Results

The clinical profiles of the 131 patients with various degrees of CKD in the present study are summarized in Table 1. The sex distributions, levels of serum albumin, prevalence of nephrotic syndrome (NS), and the clinical use of CaCO_3_ as a phosphate binder were comparable among the five CKD groups, while the differences for the rest of the parameters among the five groups of CKD were greater than would normally be expected by chance alone. The main causes of CKD were chronic glomerulonephritis in 80 patients (61%) (including biopsy-proven IgA nephropathy [n = 40]), diabetic nephropathy in 21 patients (16%), hypertensive nephrosclerosis in 13 patients (10%), lupus nephritis in five patients (4%), interstitial nephritis in three patients (2%), and the cause in the remaining nine patients was uncertain or under investigation. There were 25 patients (19%) with diabetes.

**Table 1:** Clinical profiles of CKD patients

		**CKD stages**					
	**All**	**1**	**2**	**3**	**4**	**5**	**p value**
**Clinical and laboratory characteristics**							
Patients, *n* (M/F)	131 (71/60)	18 (6/12)	27 (17/10)	29 (15/14)	18 (11/7)	39 (22/17)	p = 0.348
Age (years)	56 ± 18	36 ± 16	53 ± 18**	55 ± 16**	68 ± 16**	64 ± 15**	p < 0.001
Serum creatinine (mg/dl)	2.7 ± 2.8	0.5 ± 0.1	0.8 ± 0.2	1.2 ± 0.3	2.5 ± 0.6**	6.5 ± 2.7**	p < 0.001
eGFR (ml/min/1.73 m^2^)	46.3 ± 37.5	112.8 ± 19.5	74.7 ± 9.4**	46.7 ± 8.6**	19.9 ± 3.8**	7.9 ± 3.0**	p < 0.001
Urinary excreated protein (g/day)	2.8 ± 2.1	2.6 ± 1.3	2.2 ± 1.5	2.8 ± 1.7	4.3 ± 3.8*	2.5 ± 2.2	p = 0.039
Urinary excreted albumin (g/day)	1.9 ± 1.0	1.2 ± 0.9	1.5 ± 0.9	1.7 ± 1.1	3.1 ± 2.2*	1.5 ± 1.1	p = 0.017
Serum total protein (g/dl)	6.1 ± 0.9	6.1 ± 1.2	6.2 ± 0.2	6.5 ± 0.9	5.5 ± 1.2	6.2 ± 0.7	p = 0.023
Serum albumin (g/dl)	3.1 ± 0.8	3.1 ± 0.9	2.9 ± 0.8	3.2 ± 0.7	2.6 ± 0.9	3.1 ± 0.7	p = 0.101
Serum calcium (mg/dl)	8.6 ± 0.9	8.9 ± 0.7	8.7 ± 0.9	8.9 ± 0.5	8.2 ± 0.9*	8.2 ± 0.9*	p < 0.001
Serum phosphorus (mg/dl)	3.9 ± 1.3	3.1 ± 0.7	3.1 ± 0.5	3.12 ± 0.5	4.1 ± 1.3	5.2 ± 1.4*	p < 0.001
Serum FGF23 (pg/ml)	55.3 (37.1-217)	43.8 (36.7-63.1)	37.1 (32.0-44.0)	47.9 (36.6-62.4)	109.6 (36.0-176.0)	361.8 (228.5-617.5)*	p < 0.001
Serum iPTH (pg/ml)	53.0 (32.0-152.0)	28.0 (17.5-40.0)	33.0 (26.0-44.0)	46.0 (35.0-56.0)	87.0 (35.5-160.5)*	188 (116.0-289.0)*	p < 0.001
Serum calcitriol (pg/ml)	40.1 ± 26.0	57.1 ± 26.2	59.8 ± 23.9	52.2 ± 23.9	23.1 ± 9.5**	17.6 ± 9.4**	p < 0.001
Prevalence of NS No.(%)	34 (26)	3 (17)	5 (19)	6 (21)	8 (44)	12 (31)	p = 0.225
**Medications,*n*(%)**							
Anti-hypertensive agent(s)	67 (51)	4 (36)	11 (41)	5 (17)	10 (56)	37 (95)**	p < 0.001
Diuretic(s)	35 (27)	1 (6)	4 (15)	7 (24)	5 (28)	18 (49)**	p = 0.008
CaCO_3_ as an phosphate binder	3 (2)	0 (0)	0 (0)	0 (0)	0 (0)	3 (8)	p = 0.124
Active Vitamin D sterol (calcitriol or alfacalcidol)	9 (7)	0 (0)	0 (0)	0 (0)	1 (6)	8 (21)*	p = 0.002
Erythropoietin	30 (23)	0 (0)	0 (0)	0 (0)	2 (11)	28 (72)**	p < 0.001

*p < 0.05 vs CKD stage I, **p < 0.01 vs CKD stage I.

**Figure 1:**
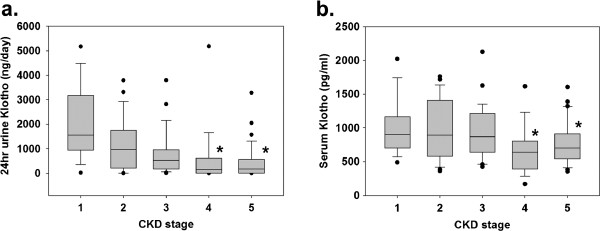
**Box plots of the amount of 24 hr urinary excreted Klotho (a) and the serum Klotho levels (b) by CKD stages.** The lower border of the box and the upper border of the box represent the first quartile and third quartile, respectively. The whiskers indicate 1.5 times the IR above and below the 25^th^ and 75^th^ percentiles. Outliers (values >1.5 times the IR) are represented by closed circles. *p < 0.05 vs CKD stage I.

**Figure 2:**
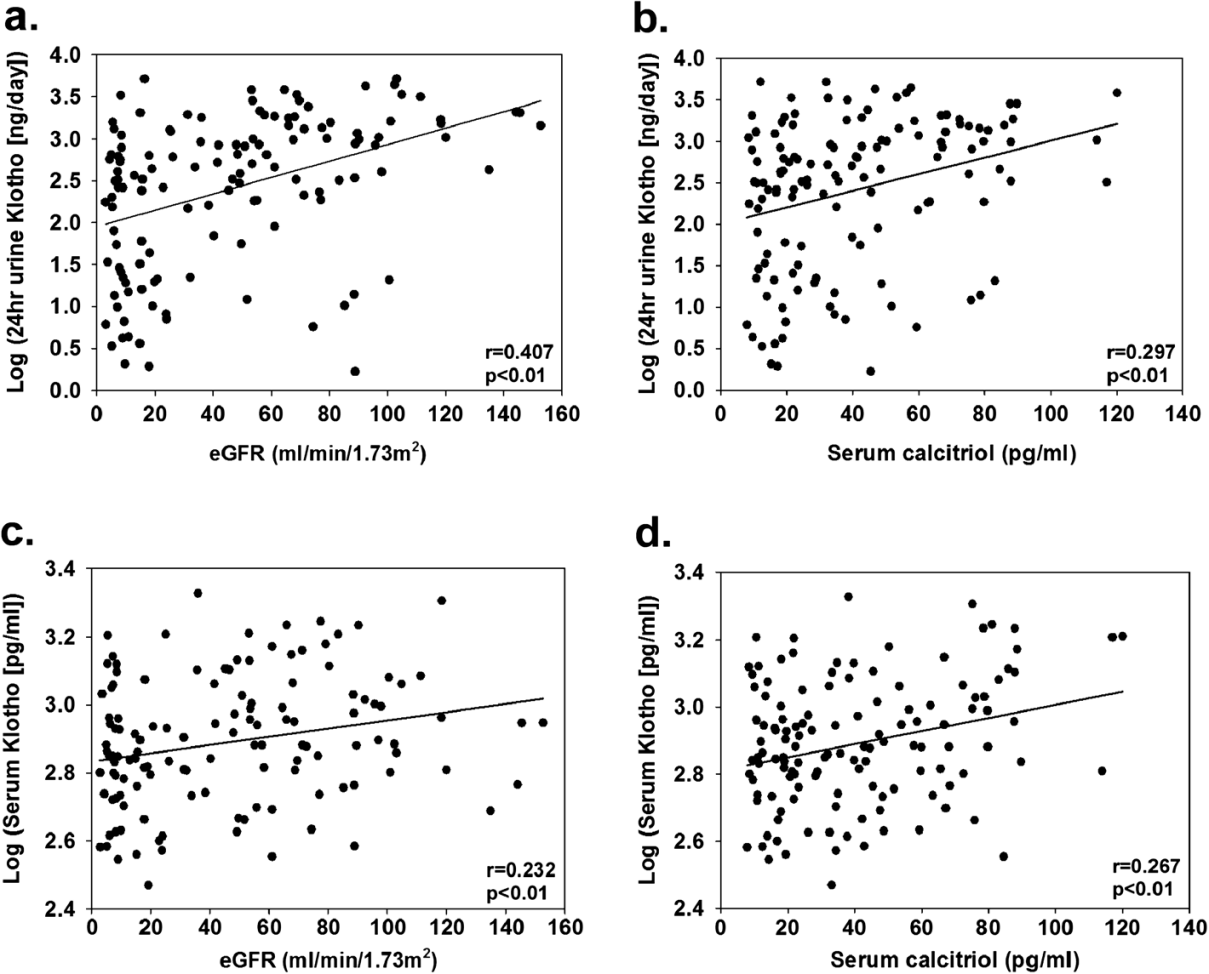
**Not only the urinary excreted Klotho but also the serum Klotho associated with both eGFR and serum calcitriol.** The log transformed value of the amount of 24 hr urinary excreted Klotho significantly correlated with the eGFR (**a**) and serum calcitriol (**b**). There were significant associations between the log transformed serum Klotho levels and the eGFR (**c**) and between the log-transformed serum soluble Klotho levels and serum calcitriol as well (**d**).

Soluble Klotho was detectable in both the urine and serum of our CKD subjects. The amount of urinary excreted Klotho during the 24 hr period ranged from 1.6 to 5178 ng/day (median 427 ng/day; IR 56.8-1293.1), and the serum Klotho concentration ranged from 163.9 to 2123.7 pg/ml (median 759.7 pg/ml; IR 579.5-1069.1). No significant differences in these values between the male and female subjects were observed. The amount of 24 hr urinary excreted soluble Klotho trended towards lower values with advancing stages of CKD (overall p < 0.001). Only the amounts of urinary excreted soluble Klotho at CKD stages 4 and 5 were significantly lower compared with CKD stage 1 (Figure 1A), while an inverse correlation between the amount of urinary excreted soluble Klotho and stages of CKD was confirmed (Spearman’s rho value = −0.462, p < 0.01). The serum soluble Klotho levels were statistically different among five CKD groups (overall p = 0.009) (Figure 1B); also, an inverse correlation was observed between the levels of serum soluble Klotho and the stages of CKD (Spearman’s rho value = −0.252, p < 0.01). The log-transformed value of the amount of 24 hr urinary excreted Klotho significantly correlated with the eGFR and serum calcitriol (Figure 2a, 2b). It correlated with the amount of urinary excreted total protein (r = 0.249, p < 0.01) and albumin (r = 0.250, p < 0.01) found in the 24 hr urine collection, serum total protein (r = −0.235, p < 0.01), serum albumin (r = −0.182, p = 0.0374), serum phosphorus (r = −0.329, p < 0.01), serum iPTH (r = −0.234, p < 0.01), and age (r = −0.304, p < 0.01), as well, but not with serum calcium and that of FGF23. The log-transformed serum Klotho concentration did not correlate with the 24 hr urinary excreted protein, and total protein, albumin, calcium, phosphorus, FGF23, and iPTH found in the serum but it did correlate with the eGFR (Figure 2c), serum calcitriol (Figure 2d), and age (r = −0.172, p < 0.05). The serum calcitriol levels correlated with eGFR (r = 0.612, p < 0.01), and they were negatively associated with iPTH (r = −0.426, p < 0.01) and FGF23 (r = −0.433, p < 0.01). A significant association between the log-transformed value of the amount of 24 hr urinary excreted Klotho and that of the serum soluble Klotho levels (r = −0.403, p < 0.01) was also observed.

A stepwise multiple regression analysis identified the eGFR (ß = 0.482) and 24-hr urinary protein (ß = 0.351) as predictive variables for the log-transformed value of the amount of 24 hr urinary excreted Klotho (Total R^2^ = 0.284, p < 0.001), while only the serum calcitriol (ß = 0.267) was selected as an explanatory factor for the serum Klotho concentration (Total R^2^ = 0.0713, p < 0.01). The log-transformed value of the amount of 24 hr urinary excreted Klotho in the 97 CKD subjects without NS correlated with the eGFR (r = 0.506, p < 0.01), serum iPTH (r = −0.225, p = 0.0264), serum calcitriol (r = 0.424, p < 0.01), and age (r = −0.348, p < 0.01); while the log-transformed serum Klotho concentration correlated with the eGFR (r = 0.263, p < 0.01) and serum calcitriol (r = 0.337, p < 0.01). Only the eGFR was included as a predictive variable for the log-transformed value of the amount of 24 hr urinary excreted Klotho (ß = 0.506, Total R^2^ = 0.256, p < 0.01), and serum calcitriol was included as a predictive variable for the log-transformed serum Klotho concentration (ß = 0.337, Total R^2^ = 0.113, p < 0.01).

**Figure 3:**
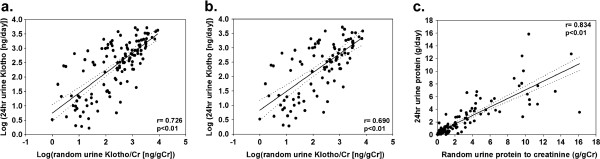
**The relationships between the log transformed value of the 24 hr urinary excreted Klotho level and that of the urine Klotho-to-creatinine ratio in all subjects (a) and in the subjects without NS (b), and between the 24 hr urine protein and the random urine protein-to creatinine ratio (c).** Note that there was a strong linear dependence between the 24 hr urine protein and the random urine protein- to-creatinine ratio. Solid line: linear regression, dotted lines: 95% confidence intervals.

**Figure 4:**
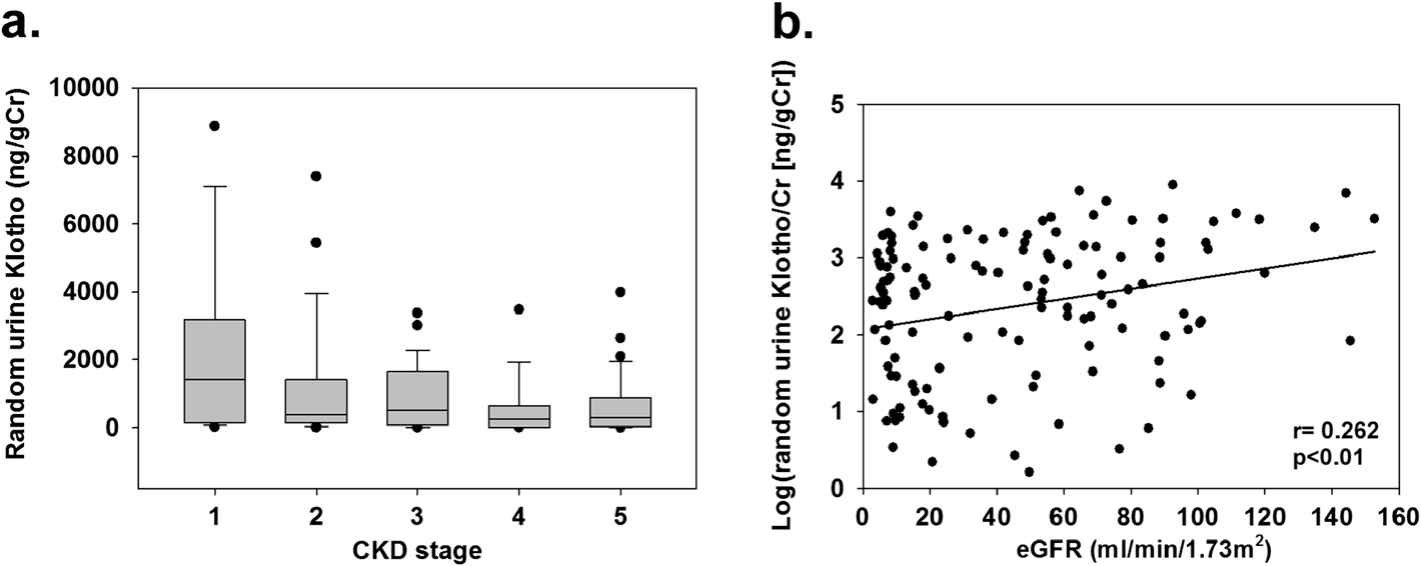
**Box plots of the random urine Klotho/Cr by CKD stages and the relationship between the log-transformed random urine Klotho/Cr and eGFR.** (**a**) No trend towards the lower values of the random urine Klotho/Cr with advancing stages of CKD was statistically confirmed. The lower border of the box and the upper border of the box represent the first quartile and third quartile, respectively. The whiskers indicate 1.5 times the IR above and below the 25^th^ and 75^th^ percentiles. Outliers (values >1.5 times the IR) are represented by closed circles. (**b**) A significant association between the log-transformed random urine Klotho/Cr and the eGFR was observed.

Regardless of the presence or absence of NS, the log-transformed value of the amount of 24 hr urinary excreted Klotho was significantly correlated with that of the random urine Klotho-to-creatinine ratio (Klotho/Cr), and no significant difference was observed between the r values among the overall patient population and the patients without NS (z = 0.53, p = 0.5947) (Figures 3a, 3b). The correlation coefficient for the linear relationship between the amount of urinary excreted protein found in the 24 hr urine collection and the random urine protein-to-creatinine ratio (Figure 3c) was significantly different from that of the linear dependence between the log-transformed value of the amount of 24 hr urinary excreted Klotho and the random urine Klotho/Cr ratio among the overall patients (z = 2.3, p = 0.0212) and the patients without NS (z = 2.6, p = 0.0093). The random urine Klotho/Cr did not trend towards lower values with advancing stages of CKD (overall p = 0.058) (Figure 4a); however, the random urine Klotho/Cr, as well as the amount of 24 hr urinary excreted Klotho, correlated inversely with the stages of CKD (Spearman’s rho value = −0.234, p < 0.01). The log-transformed random urine Klotho/Cr was also associated with the eGFR (Figure 4b); however, the log-transformed value of the amount of 24 hr urinary excreted Klotho (ß = 0.407), but not that of random urine Klotho/Cr, was identified as a predictive variable for the eGFR (Total R^2^ = 0.166, p < 0.01).

## Discussion

The serum levels of soluble Klotho may be determined by the level of FGF23, age, and renal function; however, the current data are inconclusive, probably due to the varied population of the subjects included in the studies [6,9,10,16-19]. Moreover, there has been insufficient data about the impacts of such parameters on the amount of urinary excreted soluble Klotho among human subjects with different disease status [6]. Although a decrease in urinary Klotho levels in a relatively small number of CKD patients was reported previously [20], our study clearly demonstrated for the first time that not only the serum Klotho concentration, but also the amount of 24 hr urinary excreted Klotho correlated with the eGFR among the patients with various degrees of CKD. Besides the finding that the serum Klotho concentration was negatively correlated with age, which is consistent with previous findings [9,10], we also confirmed that there was a similar trend between the amount of urinary excreted Klotho and the patient age. However, the clinical significance of age on the serum and urinary Klotho levels among the overall patients with CKD should be interpreted carefully, since our results demonstrated that the eGFR, but not age, was one of the pivotal predictive variables for the amount of urinary excreted Klotho in various CKD patients, while only the serum calcitriol was identified as a predictor of the serum soluble Klotho levels.

The renal upregulation of the Klotho gene expression by exogenous calcitriol has been reported [21], while Klotho itself has been demonstrated to play a role in the regulation of calcitriol synthesis [22]. Therefore, it is not surprising that the kinetics of soluble Klotho might also be modulated by oral calcitriol or alfacalcidol, at least in part, in some subsets of CKD patients in the current study. However, most of the serum calcitriol levels of such patients were below the normal range (data not shown), which might be consistent with previous studies [23,24]. Serum calcitriol was still included as a predictive variable for the log-transformed serum Klotho concentration when the nine patients who were treated with oral vitamin D sterols were excluded (data not shown), thus suggesting that the significance of exogenous active vitamin D sterols on the levels of serum soluble Klotho should be trivial among the patients in this study.

The renal synthesis of calcitriol, which is an active form of vitamin D, is tightly regulated by serum levels of PTH, calcium, and phosphorus [25]. FGF23, secreted from the bone, also suppresses the production of calcitriol at the level of renal proximal tubules [25,26]. Lack of data on the levels of serum 25-hydroxyvitamin D, which is the relevant indicator of exogenous supplies of vitamin D [25], precludes us from evaluating the biological significance of overall vitamin D metabolism on the kinetics of soluble Klotho among patients with CKD included in the current study. Nevertheless, the inclusion of serum calcitriol as a predictive variable for the log-transformed serum Klotho concentration (but not for the log-transformed value of the amount of 24 hr urinary excreted Klotho) despite the finding that the serum levels of calcitriol negatively correlated with levels of iPTH as well as those of FGF23 led us to propose the specific role of calcitriol as a potential determinant for serum soluble Klotho levels. This concept is strongly supported by the recent demonstration of the Klotho expression in human vascular tissues, including vascular smooth muscle cells, which is reduced during the course of CKD and can be restored by exogenous calcitriol [27]. Although the biological significance of calcitriol on the serum soluble Klotho levels and the amount of 24 hr excretion of urinary soluble Klotho should be evaluated separately, the significant association between eGFR and serum calcitriol levels demonstrated in this study implies that the magnitude of functioning or viable nephrons may ultimately determine the kinetics of both urinary and serum soluble Klotho. On the other hand, neither the amount of 24 hr urinary excreted Klotho nor the serum soluble Klotho levels correlated with several parameters, including not only FGF23 and calcium found in the serum but also with the amount of urinary excreted sodium, calcium, potassium, and phosphorus (data not shown), suggesting that the intrinsic role of the soluble Klotho on the disruption of mineral metabolism associated with various degrees of CKD might be marginal [14,28].

In our previous study with the peritoneal dialysis (PD) patients, we failed to confirm any significant associations between the amount of urinary excreted Klotho and that of albumin, which is the principal component of urinary protein in cases of glomerular proteinuria [29,30]. Therefore, we concluded that the urinary excreted Klotho detected in PD subjects is not of glomerular origin, but rather originate exclusively from the renal tubules [6]. This should be the case for the patients without NS in this study as well. Indeed, the current study demonstrated that the urinary Klotho level was significantly associated with both the urinary total protein and the albumin level among the overall CKD patients, while no such relationships were observed when the patients with NS were excluded (data not shown). We believe that such changes might be linked to the disturbed glomerular permeability that arises from various glomerulopathies associated with NS [29,31]. Therefore, the glomerular permeation of large molecules, including soluble Klotho, should regulate the amount of urinary excreted Klotho and the serum level of Klotho by modulating the internal distribution of soluble Klotho. Our recent analyses demonstrated approximately 30-50% decreases in the serum soluble Klotho levels following retroperitoneoscopic nephrectomy in living donors [32]. Preliminarily, however, we confirmed the fact that soluble Klotho is detectable even in anephric chronic hemodialysis patients (data not shown). Moreover, the recent demonstrations of the Klotho expression in vascular tissue encouraged us to regard the vascular territory as another source of serum soluble Klotho that needs to be better characterized [27]. Apparently, further studies are required to better determine the information about the kinetics of soluble Klotho, which still remains poorly understood [16,20].

Quantitating the urinary excreted protein relies on 24 hr urine collection [33], while such procedures are cumbersome and time consuming, which may lead to a delay in evaluations and inaccurate results as a result of poor compliance [34]. In this regard, the measurement of the protein-to-creatinine ratio in single random urine specimen has been focused on as an alternative approach that can be used to avoid the need for a 24 hr urine collection [35,36]. This may not be the case with quantitating urinary excreted Klotho since our study revealed that the correlation coefficients of the linear dependence between the log-transformed values of the amount of 24-hr urinary excreted Klotho and the random urine Klotho/Cr ratios among the overall patients and the patients without NS were not comparable with those of the linear dependence between the protein-to-creatinine ratios in single random urine specimens and the amounts of excreted protein found in 24-hr urine collections, implying that the Klotho/Cr ratios in random urine specimens do not serve as reliable surrogates for the amount of 24-hr urinary excreted Klotho, regardless of the presence or absence of NS. Otherwise, variations in urinary Klotho excretion throughout the day, similar to the variation observed in the urinary protein levels in a given human subject [37], may be present. Moreover, the fact that the random urine Klotho/Cr ratios trended towards lower values with advancing stages of CKD and were significantly associated with eGFR encouraged us to pursue extensive evaluations regarding the clinical impact of measuring the random urine Klotho/Cr ratios. In this regard, whether the optimal timing of the collection of a single voided urine specimen to be used as an acceptable alternative to 24 hr urine collection should be examined in larger subjects with various stages of CKD, and is therefore currently being investigated by our group.

Although the current study provided new information regarding the urinary and serum soluble Klotho among the various degrees of CKD patients, our results should be interpreted within the context of their limitations. There are several approximations of renal function, such as the clearance of creatinine, inulin, radio-active markers or radio-contrast agents; however, these methods are all impractical for everyday use and are not widely available. In particular, the creatinine clearance exceeds the GFR as a result of the tubular secretion of creatinine in patients with severely progressed chronic renal failure [6,29]. Therefore, the use of eGFR as an surrogate index in the current study should be mandatory [6,29,38]. Furthermore, multiple linear regression analyses may not necessarily be the most appropriate procedure since the correlation coefficients cannot replace visual examination of the data with precision. Nevertheless, such procedures combined with log-transformed variables may not be exceptional [10,16,18,19]. On the other hand, the data regarding the soluble Klotho in the current study may be modified by the anti-hypertensive agents such as renin-angitensin-system (RAS) inhibitors, which has been demonstrated to be associated with the up-regulation of renal Klotho expression [39]. In our subjects, the prevalence of the patients treated with RAS inhibitors trended towards greater values with advancing stages of CKD (data not shown), thereby the distribution of CKD stages were not comparable between the subjects with RAS treatment and those without RAS treatment, and this fact preclude us to precisely evaluate the impact of such agents on the amount of urinary excreted Klotho and the serum levels of soluble Klotho. Despite these limitations, we believe our data have value, especially regarding the impact of residual renal function on the amount of urinary excreted soluble Klotho and serum soluble Klotho concentration among the CKD subjects.

## Conclusion

The magnitude of functioning nephrons should be linked to the amount of urinary Klotho rather than the serum Klotho levels among patients with CKD. The use of the random urine Klotho/Cr ratio as a surrogate for the amount of 24-hr urinary excreted Klotho needs to be evaluated more carefully.

## Competing interests

The authors declare that they have no competing interests.

## Authors’ contributions

TA and HY analyzed the data, managed the database, performed statistical analysis, and drafted the manuscript. YW performed Klotho and FGF23 measurements. AN, NO, TY, ET, KI, TK, and YM analyzed the data. KS contributed substantially to the writing of the manuscript. CI, YA, SM, MK, and EK analyzed data and contributed to the data interpretation. All authors have read and approved the final manuscript.

## Pre-publication history

The pre-publication history for this paper can be accessed here:

http://www.biomedcentral.com/1471-2369/13/155/prepub
